# The Prevalence and Functional Impact of Chronic Edema and Lymphedema in Japan: LIMPRINT Study

**DOI:** 10.1089/lrb.2018.0080

**Published:** 2019-04-17

**Authors:** Misako Dai, Gojiro Nakagami, Junko Sugama, Noriko Kobayashi, Emiko Kimura, Yoko Arai, Aya Sato, Gregoire Mercier, Christine Moffatt, Susie Murray, Hiromi Sanada

**Affiliations:** ^1^ Department of Clinical Nursing, Institute of Medical, Pharmaceutical and Health Sciences, Kanazawa University, Kanazawa, Japan.; ^2^ Department of Gerontological Nursing/Wound Care Management, Graduate School of Medicine, The University of Tokyo, Tokyo, Japan.; ^3^ Division of Care Innovation, Global Nursing Research Center, Graduate School of Medicine, The University of Tokyo, Tokyo, Japan.; ^4^ Advanced Health Care Science Research Unit, Innovative Integrated Bio-Research Core, Institute for Frontier Science Initiative, Kanazawa University, Kanazawa, Japan.; ^5^ Department of Gynecology, Hokkaido University Hospital, Sapporo, Japan.; ^6^ Department of Nursing, Aomori University of Health and Welfare, Aomori, Japan.; ^7^ Ota Memorial Hospital, Ota, Japan.; ^8^ Faculty of Nursing and Social Welfare Sciences, Fukui Prefectural University, Eiheiji, Japan.; ^9^ Département d'Information Médicale, Hôpital La Colombière, Montpellier, France.; ^10^ Nottingham Trent University, School of Social Sciences, Nottingham, United Kingdom.; ^11^ Centre for Research & Implementation of Clinical Practice, London, United Kingdom.

**Keywords:** chronic edema, lymphedema, lymphoedema, prevalence, impact

## Abstract

***Background:*** This was a part of LIMPRINT (Lymphoedema IMpact and PRevalence—INTernational), an international study aimed at capturing the size and impact of lymphedema and chronic edema in different countries and health services across the world. The purpose of this study was to clarify the prevalence and the impact of chronic edema in Japan.

***Methods and Results:*** This was a two-phase facility-based study to determine the prevalence and functional impact of chronic edema in the adult population in Japan between 2014 and 2015. The prevalence study involved a university hospital, an acute community hospital, and a long-term medical facility. The impact study involved six facilities, including two outpatient clinics in acute care hospitals (one led by a physician and the other led by a nurse), inpatient wards in two acute care hospitals, and two nursing home/long-term care facilities. Various questionnaires and clinical assessments were used to gather patient demographic data and assess the functional impact of chronic edema. The results showed that chronic edema was much more prevalent in the long-term care facility than in acute care hospitals; cellulitis episodes occurred in ∼50% of cases in the gynecologist-led outpatient clinic, even though >80.0% of patients received standard management for edema; edema was found in the trunk region, including the buttock, abdomen, and chest-breast areas, in addition to the upper and lower limbs; and subjective satisfaction with edema control was low, even though the quality-of-life scores were good.

***Conclusions:*** The prevalence of chronic edema varied according to the facility type, ranging from 5.0% to 66.1%. The edema was located in all body parts, including the trunk region. Subjective satisfaction with control of edema was poor, while general quality of life was good. This large health care issue needs more attention.

## Introduction

Both lymphedema and chronic edema have strong negative effects on not only patients' health statuses but also medical expenditures around the world, but the precise epidemiological data and its impact have not been fully elucidated. This study was a part of LIMPRINT (Lymphoedema IMpact and PRevalence—INTernational), an international study aimed at capturing the size and impact of lymphedema and chronic edema in different countries and health services across the world. Its focus is to provide evidence to support the development and reimbursement of lymphedema services. The project is coordinated by Professor Christine Moffatt from the International Lymphoedema Framework (ILF). The ILF is a UK charity, whose aim is to improve the management of chronic edema and related disorders worldwide through the sharing of expertise and resources and by supporting individual countries to develop a long-term strategy for the care and management of chronic edema. Further details of the LIMPRINT project can be obtained on the ILF website (www.lympho.org/limprint). This study used the multicenter data gathered between 2014 and 2016 through the ILF, Japan branch.

In Japan, there is only a reimbursement system under national health insurance for lymphedema management of patients diagnosed with lymphedema after the treatment of uterine cancer, uterine adnexal cancer, prostate cancer, or breast cancer with lymph node dissection.^1^ However, there is no such system for chronic edema. This is partly because of the lack of epidemiological studies on chronic edema to understand its impact on patients' health.

## Aim

The purpose of this study was to clarify the prevalence and impact of chronic edema in Japan.

### Methods

#### Study design

This was a facility-based study to determine the prevalence and functional impact of chronic edema in the adult population within the ILF, Japan. LIMPRINT in Japan was a two-phase project conducted between 2014 and 2015, which included a prevalence study and an impact study.

### Prevalence study

#### Setting

In this study, all hospitalized patients at all appropriate wards were investigated to identify patients with chronic edema (excluding children <18 years and the Department of Psychiatry) on a specific day. The facilities were a university hospital (*n* = 600, 31 medical departments), an acute community hospital (*n* = 195, 13 medical departments), and a long-term medical facility (*n* = 310, 5 medical departments).

#### Definition and assessment of chronic edema

To determine the prevalent cases of chronic edema, patients whose edema continued over 3 months based on interviews and medical chart reviews were defined as having chronic edema. First, the chief investigators in cooperation with in-charge nurses at each facility assessed chronic edema by inspection. If it was difficult to determine the presence of chronic edema by inspection, the AFTD-pitting test was used.^2^ AFTD is an acronym derived from the four factors used for the test: Anatomical locations of edema assessment; Force required to pit; the amount of Time; and the Definition of edema.

#### Analysis

The prevalence of chronic edema was calculated by dividing the number of patients with chronic edema by the total number of inpatients, and 95% confidence intervals (CIs) were also calculated.

### Impact study

#### Setting

Six facilities, including two outpatient clinics in acute care hospitals (one led by a physician and the other led by a nurse), inpatient wards in two acute care hospitals, and two nursing home/long-term care facilities, participated in this study. The two outpatient clinics specialized in lymphedema management led by a gynecologist or a nurse certified as a lymphedema therapist. Two wards in a university hospital and a community hospital participated in the prevalence study. In the university hospital, the breast surgery department, gynecology department, and rehabilitation department follow up lymphedema patients with timely referral to a clinical nurse specialist in cancer nursing and a certified expert nurse in breast cancer care. In a community hospital, a clinical nurse specialist in cancer nursing with lymphedema therapist certification and a general nurse conduct their own outpatient clinic for lymphedema patients that sees patients once a week. The long-term care facilities do not have a special system for chronic edema management.

The patient inclusion criteria were as follows: older than 18 years; swelling for longer than 3 months; and able to understand the study as set out in the information sheet and give informed consent. The patient exclusion criteria were as follows: unwilling or unable to participate for whatever reason; receiving end-of-life care; and not considered to be in the patient's best interest to participate, as decided by the lead clinician.

#### Data collection

A random sample could be obtained in two facilities; the two wards from the university hospital and the community hospital with chronic edema were identified due to limited resources. A random permuted block design allowed for a one third sample to be taken. In the long-term care facility, the investigators collected data from all participants. In the outpatient clinic, participants who had an appointment for the service on that day were included in the survey.

#### Questionnaire survey

Questionnaires developed by the ILF were translated into Japanese followed by back translation to English for validation. The core tool was used to gather patient demographic data, and module tools were used to collect data on various aspects of the patients. The module tools assessed the functional impact of chronic edema and required completion through contact with the patient and clinical assessment where required. The module tools included demographic and disability (World Health Organization Disability Assessment Schedule 2.0 [WHODAS 2.0]), Quality of Life (QOL; Lymphoedema Quality of Life Study [LYMQOL] and EuroQol 5 Dimension [EQ-5D]), and details of swelling, wounds, and cancer.

WHODAS 2.0, a generic assessment instrument for health and disability, was used to assess six domains of functioning, including cognition, mobility, self-care, getting alone, life activities, and participation, with four possible response options (0 = none, 1 = mild difficulty, 2 = moderate difficulty, 3 = severe difficulty, and 4 = extreme difficulty or cannot do). The overall functioning score was calculated according to the guideline provided by the World Health Organization (WHO).^3^ The scores for each item were summed up, and then the total score was divided by 48. A higher score indicates a more severe disability status.

The EQ-5D, which is a generic health-related QOL profile instrument developed for measuring utility, was used.^4^ It contains five domains: mobility, self-care, usual activities, pain/discomfort, and anxiety/depression. A single answer with three possible response options (1 = no problem, 2 = some/moderate problems, and 3 = extreme problems) was required. The EQ-5D has been found to be sensitive to the effect of lymphedema on health related quality of life.^5^ The Japanese version was validated for the Japanese general population.^6^ Scores from the five domains were combined into a single utility score between −0.594 (worst possible state) and 1.000 (best possible state) based on the Japanese weighting system.^7^ The perceived current health state is measured by asking respondents to indicate their current health state on a Visual Analogue Scale with endpoints labeled 0 “Worst imaginable health state” and 100 “Best imaginable health state.”

The LYMQOL was used to determine the level of QOL related to lymphedema.^8,9^ This scale was developed to assess condition-specific QOL of patients with lymphedema of the limbs. The questions cover four domains (symptoms, body image/appearance, function, and mood) with four possible response options (1 = not at all, 2 = a little, 3 = quite a bit, and 4 = a lot). Scores for each domain were calculated according to the previous article.^8^ A higher LYMQOL score indicates a lower QOL. For overall QOL related to lymphedema, the responder can pick one item from 0 ( = poor) to 10 ( = excellent).

#### Analysis

Data were analyzed according to the four types of facilities involved: a gynecologist-led outpatient clinic, a lymphedema therapist nurse-led outpatient clinic, an acute care hospital ward, and a long-term care facility. Descriptive data are expressed as *N* (%) for categorical variables and medians (interquartile range) for continuous variables. The prevalence study determined the point prevalence in each facility. In the impact study, the data are presented according to the facility type. The facilities were classified into four groups: outpatient clinic, inpatient ward, nursing home, and long-term care facilities.

#### Ethical considerations

The study protocol was approved by the Medical Ethics Committee of Kanazawa University. Informed consent was obtained from each of the patients or their proxies.

## Results

### Prevalence of chronic edema

The prevalence of chronic edema was 5.0% (95% CI: 3.2%–6.8%; 30/600) in the university hospital, 7.7% (95% CI: 3.8%–11.6%; 15/195) in the acute community hospital, and 66.1% (95% CI: 60.9%–71.4%; 205/310) in the long-term medical facility; the median ages were 67.7, 70.2, and 87.2 years, respectively.

### Impact of chronic edema

In total, 111 patients were investigated for the impact of chronic edema, and the data were analyzed in each facility, the gynecologist-led outpatient clinic (*n* = 51), the lymphedema therapist nurse-led outpatient clinic (*n* = 20), the acute care hospital ward (*n* = 10), and the long-term care facility (*n* = 30).

The median patient age was 65 years, with over 95% of outpatients being female in both facilities. Inpatients of the acute care hospital ward were 59.5 years of age, with 60% female, and those at the long-term care facility were 85 years of age, with 76.7% female. In addition, 0% of outpatients and 96.7% of long-term care facility residents were immobile (Table 1).

**Table 1.** Characteristics of Participants in Each Facility

	*Outpatients*			
	*Acute care hospital*		*Inpatients*	
	*Physician led* (n = 51)	*Nurse led* (n = 20)	*Acute care hospital* (n = 10)	*Long-term care facility* (n = 30)
Age (years)	72 (68–74)	68 (59–74)	59.5 (50–72)	85 (80–90)
Sex				
Male	0 (0.0)	1 (5.0)	4 (40.0)	7 (23.3)
Female	51 (100.0)	19 (95.0)	6 (60.0)	23 (76.7)
BMI (kg/m^2^)				
<18.5	2 (3.9)	0 (0.0)	3 (30.0)	6 (20.0)
18.5 to <25.0	30 (58.8)	12 (60.0)	5 (50.0)	23 (76.7)
25.0 to <30.0	14 (27.5)	8 (40.0)	2 (20.0)	1 (3.3)
≥30.0	5 (9.8)	0 (0.0)	0 (0.0)	0 (0.0)
Upper limb mobility				
Full range of movement	50 (98.0)	17 (85.0)	9 (90.0)	14 (46.7)
Limited range of movement	1 (2.0)	1 (5.0)	0 (0.0)	1 (3.3)
No function	0 (0.0)	2 (10.0)	1 (10.0)	0 (0.0)
Lower limb mobility				
Walks unaided	50 (98.0)	20 (100.0)	5 (50.0)	6 (20.0)
Walks with aid	1 (2.0)	0 (0.0)	1 (10.0)	13 (43.3)
Chair bound	0 (0.0)	0 (0.0)	3 (30.0)	8 (26.7)
Bed bound	0 (0.0)	0 (0.0)	1 (10.0)	3 (10.0)
Immobility	0 (0.0)	0 (0.0)	2 (20.0)	29 (96.7)
Comorbidity				
Diabetes mellitus	4 (7.8)	3 (15.0)	2 (20.0)	7 (23.3)
Heart failure and/or ischemic heart disease	0 (0.0)	1 (5.0)	1 (10.0)	3 (10.0)
Neurological disorder	2 (3.9)	1 (5.0)	0 (0.0)	1 (3.3)
Peripheral arterial disease	0 (0.0)	3 (15.0)	0 (0.0)	0 (0.0)

*N* (%), median (interquartile range).

BMI, body mass index.

Lymphedema conditions in each facility are shown in Table 2. Except for two cases, they all had secondary lymphedema. Duration of edema was 5–10 years in 23 cases (46%) in the gynecologist-led clinic and 5 cases (25.0%) in the nurse-led clinic, and the duration was 3–6 months in 7 cases (70.0%) in the acute care hospital, while the duration of chronic edema ranged from 36 months to over 10 years in the patients in the long-term care medical facility. Overall, 25 cases (50%) had a history of cellulitis, with 2 cases (3.9%) having 2 episodes and 2 cases (3.9%) having 3 episodes in the gynecologist-led clinic. In each outpatient clinic, over 80% of patients received standard lymphedema care, including skin care advice, massage, multilayer garment, and exercise advice. Positive subjective opinions regarding the quality of edema control ranged from 43.3% to 50.0% in all facilities.

**Table 2.** Condition and Management of Chronic Edema and Lymphedema in Each Facility

	*Outpatients*			
	*Acute care hospital*		*Inpatients*	
	*Physician led (*n* = 51)*	*Nurse led (*n* = 20)*	*Acute care hospital (*n* = 10)*	*Long-term care facility (*n* = 30)*
Classification of edema				
Primary lymphedema	2 (3.9)	0 (0.0)	0 (0.0)	0 (0.0)
Secondary lymphedema	49 (96.1)	18 (90.0)	4 (40.0)	0 (0.0)
Unknown	0 (0.0)	2 (10.0)	6 (60.0)	30 (100.0)
Secondary swelling due to cancer				
Breast cancer	13 (25.5)	10 (50.0)	3 (30.0)	—
Endometrial/cervical cancer	24 (47.1)	8 (40.0)	0 (0.0)	—
Gastric/liver/colorectal cancer	2 (3.9)	0 (0.0)	1 (10.0)	—
Others	12 (23.5)	2 (10.0)	6 (60.0)	—
Treatment of lymphatic obstruction	49 (96.1)	20 (100.0)	4 (40.0)	0 (0.0)
Duration of edema				
3–6 Months	1 (2.0)	1 (5.0)	7 (70.0)	4 (13.3)
6 Months to 1 year	1 (2.0)	3 (15.0)	2 (20.0)	8 (26.7)
1–2 Years	2 (3.9)	2 (10.0)	0 (0.0)	3 (10.0)
2–5 Years	9 (17.6)	5 (25.0)	0 (0.0)	4 (13.3)
5–10 Years	23 (45.1)	5 (25.0)	1 (10.0)	4 (13.3)
>10 Years	15 (29.4)	4 (20.0)	0 (0.0)	7 (23.3)
Cellulitis	25 (49.0)	3 (15.0)	3 (30.0)	4 (13.3)
Infection	9 (17.6)	2 (10.0)	1 (10.0)	4 (13.3)
Hospitalization due to cellulitis	0 (0.0)	1 (5.0)	1 (10.0)	1 (3.3)
Infection, number of times				
0	42 (82.4)	18 (90.0)	9 (90.0)	26 (86.7)
1	5 (9.8)	2 (10.0)	0 (0.0)	3 (10.0)
2	2 (3.9)	0 (0.0)	1 (10.0)	0 (0.0)
3	2 (3.9)	0 (0.0)	0 (0.0)	1 (3.3)
Skin care advice	48 (94.1)	19 (95.0)	3 (30.0)	0 (0.0)
Wound dressing use	0 (0.0)	0 (0.0)	0 (0.0)	1 (3.3)
Antibiotic use	13 (25.5)	0 (0.0)	0 (0.0)	1 (3.3)
Massage	51 (100.0)	16 (80.0)	1 (10.0)	11 (36.7)
Physiotherapy	0 (0.0)	0 (0.0)	0 (0.0)	13 (43.3)
Compression garment	49 (96.1)	20 (100.0)	4 (40.0)	0 (0.0)
Multi-layer bandage	34 (66.7)	15 (75.0)	1 (10.0)	0 (0.0)
Pneumatic compression pumps	0 (0.0)	0 (0.0)	1 (10.0)	1 (3.3)
Debulking lymphedema (lymphatic surgery)	7 (13.7)	1 (5.0)	0 (0.0)	0 (0.0)
Main categories of treatment within Complex Decongestive Therapy				
Exercise advice	40 (80.0)	17 (85.0)	1 (0.0)	0 (0.0)
Cellulitis advice	49 (98.0)	19 (95.0)	1 (0.0)	0 (0.0)
Psychological support	43 (86.0)	10 (50.0)	0 (0.0)	0 (0.0)
Subjective control of chronic edema: in your opinion, is the swelling well controlled?	23 (46.0)	10 (50.0)	5 (50.0)	13 (43.3)

*N* (%).

The relevant anatomical locations of chronic edema for both sides of the whole body are summarized in Table 3. Of all the body parts, chronic edema was most common in the lower limb, foot, lower leg, and upper leg. Table 4 shows lymphedema status for outpatients in acute care hospitals. Upper lymphedema patients at International Society of Lymphology (ISL) stage II accounted for 69.2% of cases in the gynecologist-led and 70.0% in the nurse-led outpatient clinics. Lower lymphedema patients at ISL late stage II accounted for 63.2% of cases in the gynecologist-led and 40.0% in the nurse-led outpatient clinics. There were no wounds in the affected edema sites in these subjects.

**Table 3.** Anatomical Locations of Chronic Edema for Both Sides of the Whole Body

	*Outpatients*			
	*Acute care hospital*		*Inpatients*	
	*Physician led (*n* = 102)*	*Nurse led (*n* = 40)*	*Acute care hospital (*n* = 20)*	*Long-term care facility (*n* = 60)*
Head, neck, and face	0 (0.0)	0 (0.0)	0 (0.0)	0 (0.0)
Upper limbs				
Fingers	10 (9.8)	8 (20.0)	1 (5.0)	1 (1.7)
Hand	10 (9.8)	8 (20.0)	2 (10.0)	2 (3.3)
Lower arm	13 (12.7)	10 (25.0)	3 (15.0)	3 (5.0)
Upper arm	12 (11.8)	11 (27.5)	1 (5.0)	2 (3.3)
Shoulder	4 (3.9)	8 (20.0)	0 (0.0)	1 (1.7)
Lower limbs				
Toes	31 (30.4)	4 (10.0)	5 (25.0)	11 (18.3)
Foot	40 (39.2)	9 (22.5)	13 (65.0)	28 (46.7)
Lower leg	41 (40.2)	12 (30.0)	14 (70.0)	23 (38.3)
Upper leg	43 (42.2)	11 (27.5)	10 (50.0)	3 (5.0)
Trunk				
Buttock	21 (20.6)	4 (0.0)	4 (20.0)	0 (0.0)
Abdomen	20 (19.6)	0 (0.0)	4 (20.0)	0 (0.0)
Upper chest-breast	2 (2.0)	0 (0.0)	0 (0.0)	0 (0.0)
Genital area (vulva, scrotum, and penis)	2 (2.0)	0 (0.0)	0 (0.0)	0 (0.0)

*N* (%), numbers show both sides of each body part. Physician: gynecologist.

**Table 4.** Lymphedema Status of Outpatients in Acute Care Hospitals

	*Physician led*		*Nurse led*	
	*Upper lymphedema (*n* = 13)*	*Lower lymphedema (*n* = 38)*	*Upper lymphedema (*n* = 10)*	*Lower lymphedema (*n* = 10)*
Stemmers sign				
Present	2 (15.4)	20 (52.6)	7 (70.0)	5 (50.0)
Absent	11 (84.6)	18 (47.4)	3 (30.0)	5 (50.0)
ISL classification				
ISL stage I	2 (15.4)	1 (2.6)	0 (0.0)	0 (0.0)
ISL stage II	9 (69.2)	11 (28.9)	7 (70.0)	4 (40.0)
ISL stage late II	2 (15.4)	24 (63.2)	3 (30.0)	4 (40.0)
ISL stage III	0 (0.0)	2 (5.3)	0 (0.0)	1 (10.0)

*N* (%).

ISL, International Society of Lymphology.

Table 5 shows the generic and disease-specific QOL status assessed by WHODAS 2.0, EQ-5D, and LYMQOL for the upper and lower limbs, respectively.

**Table 5.** Disease-Specific Quality-of-Life Status in Acute Care Hospitals

	*Outpatients*		
	*Physician led (*n* = 51)*	*Nurse led (*n* = 20)*	*Inpatients (*n* = 10)*
WHODAS 2.0	0.0 (0.0–10.4)	2.1 (0.0–3.1)	58.3 (37.5–72.9)
EQ-5D			
Utility score	1.000 (0.796–1.000)	0.796 (0.796–1.000)	0.516 (0.312–1.000)
Perceived health status	88.0 (88.0–100.0)	100 (99.6–100.0)	71.7 (60.0–100.0)
LYMQOL: upper limb	*n* = 13	*n* = 10	*n* = 1
Function	1.2 (1.0–2.2)	1.45 (1.2–2.0)	2.0
Appearance	1.8 (1.0–2.6)	1.7 (1.4–2.2)	1.4
Symptom	2.3 (1.7–2.7)	1.9 (1.5–2.3)	3.0
Mood	1.0 (1.0–2.0)	1.9 (1.0–2.5)	2.8
Overall	8.0 (7.0–9.0)	7.25 (6.0–8.0)	4.0
LYMQOL: lower limb	*n* = 38	*n* = 10	*n* = 9
Function	1.6 (1.1–2.3)	1.5 (1.1–2.0)	2.8 (2.5–2.8)
Appearance	2.3 (1.2–2.8)	2.2 (1.3–2.8)	2.2 (2.0–2.3)
Symptom	2.0 (1.3–2.7)	1.9 (1.7–2.2)	—
Mood	2.0 (1.7–2.3)	1.3 (1.0–2.0)	2.8 (2.5–3.0)
Overall	8.0 (6.0–8.0)	7.5 (6.0–8.0)	5.0 (4.0–6.0)

Median (interquartile range).

## Discussion

There were four new findings in this study. First, the prevalence of chronic edema was much higher in the long-term care facility than in the acute care hospitals. Second, the prevalence of cellulitis episodes was ∼50% in the gynecologist-led outpatient clinic, even though over 80.0% of the patients underwent standard management for edema. Third, the edema could be found in the trunk region, including the buttock, abdomen, and chest-breast areas, in addition to the upper and lower limbs. Fourth, subjective satisfaction with control of edema was low, even though the QOL scores were good.

The prevalence of chronic edema was much higher among patients in a long-term medical facility (66.1%) with median age of 87.2 years than in both acute hospitals, including a university hospital (5.0%; median age 67.7 years) and a community hospital (7.7%; median age 70.2 years). According to the previous prevalence study of chronic edema, Moffatt et al. reported that, while chronic edema/lymphedema can occur at any age, there was a clear increase in the rate with age.^10,11^ Japan is already a super-aged society: the 2017 statistics showed an older adult population of ∼28% and an average life expectancy of ∼80.7 years in men and 87.0 years in women.^12^ In Japan, more attention to edema management for elderly people is needed.

In these results, the highest prevalence of cellulitis episodes was 49.0% in the gynecologist-led outpatient clinic compared to other facilities, ranging from 15.0% to 30.0%. In the gynecologist-led outpatient clinic, over 80.0% of patients received standard management for lymphedema, such as skin care advice (94.1%), massage (100.0%), compression garments (96.1%), exercise advice (80.0%), and cellulitis advice (98.0%), which are known as best practices.^13^ The reason why the number of cellulitis episodes was high despite standard care in the gynecologist-led clinic was the larger numbers of lower lymphedema patients (*n* = 38) and late II patients (63.2%) than in the nurse-led clinic. Lower limb lymphedema and its severity are factors related to cellulitis.^14^ Further investigation (e.g., frequency and methodology of management, and patient compliance) is needed to clarify the details that potentially prevent cellulitis episodes in these patients.

This study also showed that edema can be found in the trunk region. In the gynecologist-led outpatient clinic, chronic edema was found in the buttock, abdomen, and upper chest-breast areas. Previous studies have not provided the details of the regions affected by chronic edema in outpatient clinics. Further investigation of the details of the care provided to the chronic edema in those regions is needed.

Generic QOL scores in lymphedema outpatients were 88.8%–100.0%, a relatively good status compared to inpatients at an acute care hospital (71.7%). It is quite interesting to note that the utility score in patients at outpatient clinics was extremely high (0.796–1.000). Professional-led clinics can offer optimal options for lymphedema management that can preserve patients' functional status, leading to a high utility score. However, subjective satisfaction with control of chronic edema was only 46.6% or 50.0% in both outpatients and inpatients. These results might be explained by the fact that health-related QOL status was not directly affected by subjective satisfaction with edema control. Further study will be needed to improve these subjective satisfaction ratings.

This study has two limitations. First, this investigation was a facility-based study, not community based. Therefore, it will not be compared to other community-based studies in LIMPRINT. Second, the questionnaires related to QOL were not suitable for elderly inpatients due to cognitive dysfunction and dementia. Therefore, questionnaires for these subjects might need to be developed.

## Conclusion

This LIMPRINT Japan branch survey investigated the prevalence of chronic edema in various care settings and its impact using a detailed questionnaire. The prevalence of chronic edema varied according to the facility type, ranging from 5.0% to 66.1%. The edema was located in all body parts, including the trunk region. Subjective satisfaction with control of edema was poor, while general QOL was good. This large health care issue needs more attention.
